# Molecular profiling of frontal and occipital subcortical white matter hyperintensities in Alzheimer’s disease

**DOI:** 10.3389/fneur.2024.1470441

**Published:** 2025-01-07

**Authors:** Sulochan Malla, Annie G. Bryant, Rojashree Jayakumar, Benjamin Woost, Nina Wolf, Andrew Li, Sudeshna Das, Susanne J. van Veluw, Rachel E. Bennett

**Affiliations:** ^1^Department of Neurology, Massachusetts General Hospital, Charlestown, MA, United States; ^2^Harvard Medical School, Boston, MA, United States; ^3^School of Physics, The University of Sydney, Sydney, NSW, Australia

**Keywords:** Alzheimer’s disease, white matter hyperintensities (WMHs), brain vasculature, blood vessels, angiogenesis, heat shock proteins (HSPs), protein folding

## Abstract

White matter hyperintensities (WMHs) are commonly detected on T2-weighted magnetic resonance imaging (MRI) scans, occurring in both typical aging and Alzheimer’s disease (AD). Despite their frequent appearance and their association with cognitive decline in AD, the molecular factors contributing to WMHs remain unclear. In this study, we investigated the transcriptomic profiles of two commonly affected brain regions with coincident AD pathology—frontal subcortical white matter (frontal-WM) and occipital subcortical white matter (occipital-WM)—and compared with age-matched cognitively intact controls. Through RNA-sequencing in frontal- and occipital-WM bulk tissues, we identified an upregulation of genes associated with brain vasculature function in AD white matter. To further elucidate vasculature-specific transcriptomic features, we performed RNA-seq analysis on blood vessels isolated from these white matter regions, which revealed an upregulation of genes related to protein folding pathways. Finally, comparing gene expression profiles between AD individuals with high- versus low-WMH burden showed an increased expression of pathways associated with immune function. Taken together, our study characterizes the diverse molecular profiles of white matter changes in AD and provides mechanistic insights into the processes underlying AD-related WMHs.

## Introduction

White matter hyperintensities (WMHs) are imaging abnormalities observed on T2-weighted MRI scans (1). These abnormalities increase with age, with an estimated 90% of individuals over 65 years of age exhibiting such lesions (2, 3). WMHs may manifest across various regions of the brain, particularly in periventricular, deep frontal subcortical, and parieto-occipital subcortical white matter (WM) areas (4). Increased WMH volume is associated with cognitive deterioration and the onset of neurological conditions like Alzheimer’s disease (AD) (5). Moreover, WMH volume is predictive of both the development of mild cognitive impairment and the rate of cognitive decline (4, 6–9).

Despite these clear associations with cognitive decline, the underlying cause(s) of these WM abnormalities remain somewhat unclear (10). Factors contributing to WMH formation may include chronic cerebral hypoperfusion (11, 12), inflammation (13), microglial and endothelial cell activation (14), blood brain barrier dysfunction (15), myelin degeneration, and axonal loss (16). Genome-wide association studies indicate that genes regulating blood pressure (17) along with other risk factors such as diabetes, hypercholesterolemia, smoking, carotid artery disease, atrial fibrillation, and heart failure (18) are associated with WMHs. Considering this large vascular component, WMHs are generally considered as indicators of small vessel disease (4, 17, 19–21). On the other hand, neuropathological examinations comparing frontal and parietal WM lesions in aging and AD indicate that they may be caused by different AD-related mechanisms, such as the deposition of hyperphosphorylated tau and amyloid beta (Aβ) (3, 22–26).

Altogether, questions remain regarding whether WMHs in AD exhibit unique features compared to aged, cognitively intact controls, and whether similar processes underlie WMHs in different brain areas. To address these key questions, we performed RNA-sequencing (RNA-seq) analysis of bulk tissues and blood vessels isolated from the WM of AD cases and age-matched, cognitively intact controls. Through detailed transcriptomic characterization of WMH-susceptible regions, this study provides insights into the biological underpinnings of these lesions. It provides new evidence that, despite appearing similar by MRI, AD-related WM changes are distinct from other aging processes and the development of WMHs reflect a wide array of biological processes including vasculature and protein folding functions.

## Methods

### Human donor tissue collection

All brain tissues included in this study were obtained from the Massachusetts Alzheimer’s Disease Research Center (MADRC) with the consent of the patients or their families and approval from the Mass General Brigham Institutional Review Board (IRB; 1999P009556). Donor tissues were assessed by a neuropathologist following the NIA-AA guidelines (27). Aged, cognitively intact control cases (*n* = 5, 3F/2M) were defined as donors with low amyloid and neuritic plaque scores, Braak neurofibrillary tangle (NFT) scores of 0/I/II, and no history of dementia. Alzheimer’s disease (AD) cases (*n* = 9, 6F/3M) had high amyloid scores with moderate to frequent neuritic plaques and extensive tau pathology in the neocortex (Braak V/VI). Additionally, AD donors were selected based on the availability of *in vivo* MRI. Some participants in both the control and AD groups exhibited cerebral amyloid angiopathy (CAA) and cerebrovascular disease (CVD) upon neuropathological evaluation (MADRC) (28–30). For instance, among the 5 control participants, 4 had CVD and 1 had CAA whereas among AD participants, there were 7 with CVD and 6 with CAA. A summary of donor tissues used in this study, including AD neuropathologic change, is presented in Table 1.

**Table 1:** Participant demographic and neuropathological information.

Neuropath group	Sex	Age	ABC Score	AD Neuropath. Change	*In vivo* WMH volume (mm^3^)	WMH burden	Death-scan interval (years)	CAA	CVD
Control-1	F	81	A2, B1, C1	Low	NA	NA	NA	Yes	Yes
Control-2	F	79	A0, B1, C0	Not	NA	NA	NA	No	No
Control-3	M	89	A1, B1, C0	Not	NA	NA	NA	No	Yes
Control-4	M	>=90	A0, B1, C0	Not	NA	NA	NA	No	Yes
Control-5	F	89	A0, B1, C0	Not	NA	NA	NA	No	Yes
AD-1	F	84	A3, B3, C2	High	104	Low	4.06	Yes	No
AD-2	M	84	A3, B3. C3	High	2,258	Low	2.28	No	Yes
AD-3	F	64	A3, B3, C3	High	2,754	Low	4.87	Yes	Yes
AD-4	F	72	A3, B3, C3	High	6,469	Low	1.19	No	Yes
AD-5	M	81	A3, B3, C3	High	11,013	Low	2.39	Yes	Yes
AD-6	F	78	A3, B3, C2	High	23,654	High	0.69	Yes	Yes
AD-7	M	>=90	A3, B3, C2	High	37,387	High	4.87	Yes	Yes
AD-8	F	85	A3, B3, C2	High	40,529	High	3.31	No	No
AD-9	F	86	A3, B3, C3	High	62,279	High	2.37	Yes	Yes

Details include sex (M = male; F = female), age at death (ages > =90 have been aggregated according to de-identification standards), ABC scores and AD neuropathologic change evaluation (27), *in vivo* measures of total WMH volume, the WMH burden group assignment that was used for comparisons in this study, the interval between the *in vivo* MRI and death, and the presence or absence of comorbidities [CAA = cerebral amyloid angiopathy (30); CVD = cardiovascular disease (28, 29)] as assessed by neuropathology.

### *In vivo* MRI image acquisition and processing

Fluid-attenuated inversion recovery (FLAIR) and magnetization-prepared rapid acquisition with gradient echo (MPRAGE) images were collected using a 3T scanner (MAGNETOM Trio, Siemens Healthineers) under existing clinical research protocols approved by the Mass General Brigham IRB and shared via the Neuroimaging and Biomarker Core of the MADRC. FLAIR Images were collected using the following parameters: TE = 303 ms, TI = 2,200 ms, TR = 6,000 ms with a resolution of 1.0 × 1.0 × 1.0 mm^3^. MPRAGE used the following parameters: TE = 1.61 ms, TI = 1,100, TR = 2,530 ms with a resolution of 1.0 × 1.0 × 1.0 mm^3^. FLAIR and MPRAGE DICOM series were unpacked and converted to NIFTI volumes with *dcmunpack* from FreeSurfer (v7.1.1; https://surfer.nmr.mgh.harvard.edu/). We utilized the sequence adaptive multimodal sequencing (samseg) module from FreeSurfer (31, 32) with the lesion flag for automatic and unsupervised segmentation of WMH from the native-space FLAIR and MPRAGE volumes. Volumetric segmentation and cortical reconstruction were performed using the standard recon-all pipeline from FreeSurfer. The FLAIR and samseg-derived WMH volumes were aligned and registered to the FreeSurfer-processed MPRAGE using *bbregister* from FreeSurfer. WMH volume statistics were derived using the *mri_segstats* function from FreeSurfer after binarizing the WMH segmentation and co-registering to the FreeSurfer-processed MPRAGE using *mri_vol2vol* with the .lta file generated from bbregister. These WMH volumes are reported in Table 1.

### *Ex vivo* MRI imaging and quantification

Archived formalin-fixed coronal brain slabs (stored for up to 8 years) were rinsed for 24 h in PBS and then incubated overnight in fresh neutral buffered 10% formalin (Sigma-Aldrich, catalog # HT501128). Two slabs from each donor were selected for imaging corresponding to slices nearest the frontal and occipital horn of the lateral ventricles. Slices were stacked in neutral buffered 10% formalin, gently weighted and secured with parafilm (to eliminate bubbles and minimize tissue shifting), and imaged using a 7T MRI scanner (MAGNETOM Trio, Siemens Healthineers) equipped with a 32-channel head coil. A turbo-spin echo (TSE) sequence was acquired (TE = 50 ms, TR = 1,000 ms) with a resolution of 0.6 × 0.6 × 0.6 mm^3^ (total scan time 0.5 h). MRI files were imported to Image-J and regions of interest (ROIs) were manually drawn to isolate each slab from the stack. These images were subsequently used to guide dissection of white matter regions based on brightness relative to the surrounding tissue. Dissected tissues were then embedded in paraffin for downstream histology and immunohistochemistry.

### Histology

Paraffin-embedded frontal-WM and occipital-WM tissues from AD and adjacent control tissues were sectioned on a microtome at 7 μm, and sections were mounted on Superfrost slides as described previously (33). Slides containing paraffin-embedded tissue sections were deparaffinized and rehydrated in a sequence of solutions, including (5 min each) 100% xylene, 100% ethanol (EtOH), 95% EtOH, 70% EtOH, and distilled H_2_O. For Luxol Fast Blue staining, sections were then stained overnight at 60°C using 0.1% Luxol Fast Blue (Acros Organics, catalog # AC212170250) in 95% EtOH and 0.05% acetic acid. After staining, sections were rinsed in 95% EtOH and distilled water. Differentiation was carried out by immersing the sections in Lithium Carbonate solution (0.05%; Sigma Aldrich, catalog # L-3876) for 1 min. Subsequently, sections underwent dehydration with an ascending EtOH series followed by xylene and were cover-slipped with Eukitt Quick-Hardening Medium (Sigma, catalog # 03989).

For the validation of targets identified by transcriptomic analysis, tissue sections were deparaffinized in 100% xylene (two changes at 5 min each) followed by rehydration in descending concentrations of EtOH (two changes of 100% EtOH for 5 min followed by 95, and 80% EtOH for 1 min). Epitope retrieval was conducted in citrate buffer (pH 6.0, containing 0.05% Tween-20) using microwave heating at 95°C for 20 min (20% power). Sections were then blocked with 5% BSA in TBS containing 0.25% Triton-X for 1 h at room temperature and then incubated with primary antibodies overnight at 4°C. Primary antibodies used in this study targeted HSPA6 (Santa Cruz, catalog # sc-374589) and HSP90AA1 (Invitrogen, catalog # PA3-013). For 3,3′-diaminobenzidine (DAB) staining, sections were also pre-treated with 3% hydrogen peroxide (10 min). Following primary antibody incubation, biotinylated secondary antibodies were added for 1 h, then incubated with ABC solution (streptavidin-HRP; Vector Laboratories, catalog # PK-6100) for 1 h and finally, incubated with DAB (Sigma Aldrich, catalog # D5905) in TBS containing nickel chloride for 15 min. DAB-labeled sections were subsequently rinsed in TBS, dehydrated and cover-slipped with Eukitt. All images were obtained using an Olympus VS120 automated slide scanner and a 20× lens.

### Isolation of blood vessels from freshly frozen brain tissue

Regions corresponding to subcortical white matter near the frontal and occipital horns of the lateral ventricles were dissected from frozen tissue slabs. These regions were selected *a priori* due to their susceptibility to developing WMHs with aging and AD (10, 26, 34). A 25 mg piece of each region was reserved for bulk RNA sequencing. Blood vessels were isolated from 200 to 300 mg of frozen frontal- and occipital-WM human tissue from both the control and AD donors following the method previously described (35–37). Briefly, tissues were minced in 2 mm sections using a razor blade in ice-cold B1 buffer (Hanks Balanced Salt Solution with 10 mM HEPES, pH 7; Thermo Fisher Scientific). Subsequently, samples were manually homogenized using a 2 mL dounce homogenizer (12 strokes). The resulting homogenized mixture was transferred into a conical tube filled with 20 mL of B1 buffer and centrifuged at 2,000 *g* for 10 min at 4°C. The supernatant was discarded to obtain the pellet, which was then suspended in 20 mL of B2 buffer (B1 buffer with 18% dextran, Sigma-Aldrich) by vigorously mixing for 1 min to separate out myelin. Following this, samples were centrifuged at 4,400 *g* for 15 min at 4°C to remove the myelin layer, after which the pellet was resuspended in 1 mL of B3 buffer (B1 buffer with 1% Bovine Serum Albumin, Sigma-Aldrich). Homogenates were then filtered in a 20 mm mesh (Millipore) pre-equilibrated with 5 mL of ice-cold B3 solution. Brain blood vessels were rinsed with 15 mL of ice-cold B3 solution, detached from the filters by immersing them in 20 mL of B3 ice-cold solution, and centrifuged at 2,000 g for 5 min at 4°C. Finally, the pellet was resuspended in 1 mL of ice-cold B1 solution and re-centrifuged at 2,000 *g* for 5 min at 4°C. The supernatant was discarded to obtain the blood vessel pellet, each of which was stored at −80°C until further use.

### Bulk RNA-sequencing, analysis, and quality control

Frozen isolated blood vessels or 25 mg bulk brain tissue were placed in buffer RLT (Qiagen) and sonicated with 20 pulses at 10% power. The resulting homogenates were centrifuged at 13,000 rotations per minute (rpm) for 3 min and RNA in the supernatant was purified using the RNase Mini Kit (Qiagen). A NanoDrop spectrophotometer was used to measure the concentration of RNA in each sample. All samples were diluted to 20 ng/μl in RNase free water and sent to Discovery Life Sciences Genomics (Huntsville, AL) for RNA sequencing (RNA-seq). We performed transcript-level abundance quantification from the FASTQ sequencing files using *Salmon* (v1.5.2) in mapping-based mode (38). Briefly, the primary assembly human genome (GRCh38) and full human transcriptome were acquired from GENCODE release 36. The genome was appended to the transcriptome to create a combined “gentrome” file for indexing using *Salmon’s* index command with the gencode flag. Each sample’s paired-end FASTQ reads were then quantified against the full decoy-aware transcriptome using salmon’s *quant* function with –*validate Mappings* and –*gc Bias* flags. We set −l A so *Salmon* would automatically determine the library type. The *Salmon* transcript quantification files (quant.sf.gz) were imported into R using the *tximport* package (38, 39) with a custom constructed TxDb object created from the GENCODE GRCh38 annotation .gtf file using the *makeTxDbFromGFF* function.

To compare variations in the white matter transcriptome with neuropathological group as well as potential confounding variables like age and sex, we applied principal components analysis (PCA) to the transcripts per million (TPM) count data from the bulk white matter tissue samples in the frontal versus occipital bulk white matter tissues separately. PCA was performed using the *PCA* function from the *FactoMineR* package in R (version 2.11), with results visually inspected using a Scree plot with cumulative variance explained as well as scatterplots of principal component scores colored by neuropathological group, sex, or age.

### Analysis of differentially expressed genes (DEGs)

Differential gene expression analysis was performed using *DESeq2*, which estimates the mean–variance relationship and log-based fold changes in the count data using the negative binomial distribution (40). We constructed the *DESeqDataSet* object using the transcript abundance files and the *tximport* function, specifying the design formula as ~ Group, where the Group corresponds to AD versus CTRL. For both the AD with WMH (AD) versus control and the high- versus low-WMH AD analyses, we partitioned the data into four groups before running DESeq: (1) frontal-WM bulk tissue; (2) frontal-WM blood vessels; (3) occipital-WM bulk tissue; and (4) occipital-WM blood vessels (BV). In each comparison, we performed differential expression analysis with the *DESeq* function from the DESeq2 Bioconductor package (version 1.44.0) using all the default function parameters. Specifically, this function combines three key steps: (1) estimating size factors to normalize for sequencing depth; (2) estimating the dispersion for each gene (with a minimum mean count of 0.5) by fitting a parametric model assuming a log-linear relationship; and (3) fitting a negative binomial generalized linear model to test for differential expression per gene using the Wald test. We extracted the results from each DESeq analysis using the *results* function, with independent filtering set to FALSE. We considered a gene to be differentially expressed in either AD versus control or high- versus low-WMH comparisons if the Benjamini–Hochberg (BH)-adjusted *p* < 0.05, and magnitude of the log2 fold change (log2FC) > 0.5. The top 30 DEGs per analysis were visualized in a volcano plot by first performing a variance stabilizing transformation (*vst* function) in *DESeq2* and plotting the transformed values.

### Cell type enrichment analysis for blood vessels

To confirm the enrichment of vascular cells in isolated blood vessels, we identified the top 500 upregulated genes in control blood vessels versus bulk tissue for both frontal- and occipital-WM tissue. We quantified the cell type enrichment of these DEGs using expression-weighted cell type enrichment (EWCE) analysis, as described in Skene and Grant (41). We used the *EWCE* package from Bioconductor in R (version 3.19) and a provided murine cortex and hippocampus transcriptomic dataset as the reference, as in similar human neuropathological studies (42–46). This reference dataset includes seven coarser cell types (including endothelial cells, labeled “endothelial-mural”) which subdivide into 48 finer cell types, including four endothelial subtypes (vascular endothelial cells 1 and 2, “Vend1” and “Vend2”; vascular smooth muscle cells, “Vsmc”; amd pericytes, “Peric”). The *drop_uninformative_genes* function was used to filter to genes with 1:1 human—mouse ortholog mapping that show variance across cell types. We performed a bootstrap significance test for enrichment with 10,000 permutations using the *bootstrap_enrichment_test* function from EWCE, supplying the top 500 blood vessel DEGs as “hit” genes. We applied this test at both the coarser level 1 annotations and finer level 2 annotations to characterize cell type specificity among the 500 blood vessel DEGs. This function returns the fold enrichment of each cell type, derived by computing the expression of the target gene list divided by the average expression across randomly subsampled genes across bootstrap permutations.

### Metascape and synapse functional enrichment analysis

Gene ontology (GO) annotation and functional enrichment analysis of DEGs was carried out using Metascape^1^ with default settings as previously described (47). The Metascape multi-gene-list meta-analysis tool was used to compare common functional pathways among DEGs in AD versus control and high- versus low-WMH burden for both brain regions. For comparative functional gene enrichment annotation obtained from Metascape, DEGs with specified cut off value were submitted to ShinyGO^2^ (48). To investigate dysregulated synaptic organization, we submitted DEGs to synaptic ontology database SynGO^3^ (49). The brain expressed background gene set was used to identify the enriched synaptic components.

### Statistical analysis

Individual data points are shown in bar plots, where bar height indicates mean and error bars show ± standard deviations. The volume of *in vivo* WMH in low- versus high-WMH samples was compared using Student’s *t*-test in GraphPad Prism (*p < 0.05, **p < 0.01, ***p < 0.001). All other analysis was performed in R as indicated above.

## Results

### Characterization of WMH in control and AD donor tissues

**Figure 1:**
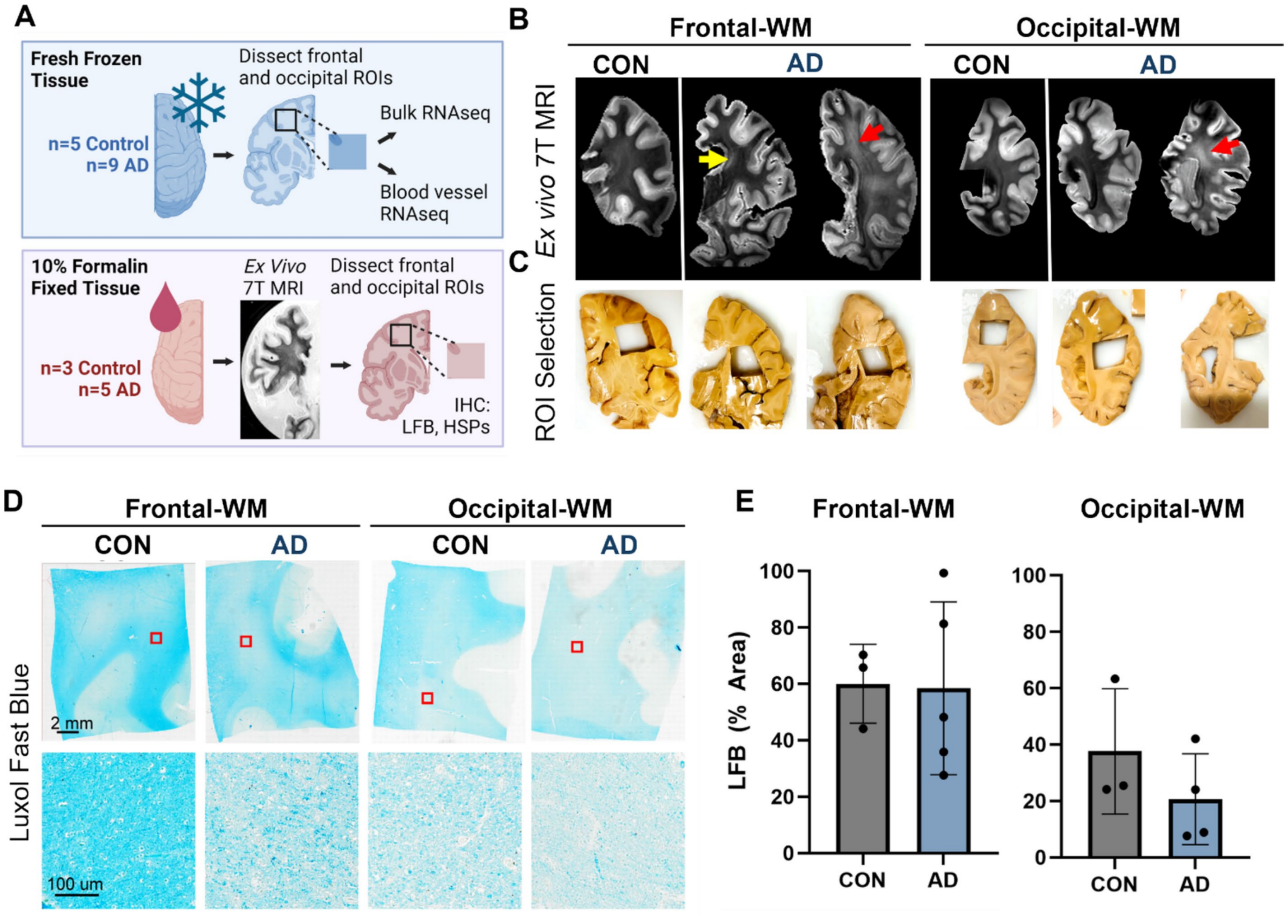
Experimental outline and characterization of WMH in control and AD cases. **(A)** Schematic representation of experimental outline and conditions. Tissues were obtained from the Massachusetts ADRC. One hemisphere of the brain was fixed in 10% formalin and was used for *ex vivo* 7T MRI and histopathology. The other hemisphere was freshly frozen, and tissues were used for RNA-seq from the bulk tissues and isolated blood vessels. Not all donors used for RNA-seq had the alternate hemisphere and corresponding brain regions available for histology. **(B)** Examples of *ex vivo* T2-weighted TSE MRI scan showing the frontal- and occipital-WM from control and AD tissues. WMHs were visible as subtle periventricular changes only (yellow arrow) or, in the most affected brains, as hyperintense areas extending into deep subcortical WM (red arrows). **(C)** Dissection of region of interest (ROI) showing WMH in the frontal- and occipital-WM as indicated by the *ex vivo* MRI images in **(B)**. **(D)** Luxol Fast Blue (LFB) staining of the control and AD samples in frontal- and occipital-WM. **(E)** The percentage area of LFB staining is shown in control and AD for frontal- and occipital-WM.

We first selected *n* = 9 AD donors who had undergone T2-weighted MRI scans during life within 5 years prior to death and matched these to *n* = 5 aged controls. After reviewing the *in vivo* MR images (Supplementary Figures S1A,B), we dissected frozen tissues from frontal- and occipital-WM for RNA-seq, as these two areas are commonly affected by WMHs (Figure 1A). In a subset for which formalin-fixed tissues were also available (*n* = 8), we performed *ex vivo* MRI of the contralateral hemisphere and observed that WM changes were similar across the aged controls and AD donors (Figure 1B). However, we note that the greatest burden of WMH was observed in 2 out of 5 AD cases in these *ex vivo* images, corresponding to what was observed *in vivo*. From these formalin-fixed tissues, we dissected additional WM regions and assessed WM integrity by Luxol Fast Blue (LFB) (Figures 1C,D). No significant difference was observed in the percent area of LFB staining within WM, indicating similar myelin status in AD and control samples (Figure 1E). In sum, the integrity of WM was overall similar between AD and aged control tissues.

### Alterations in brain vasculature pathways are underlying features of AD WM

**Figure 2:**
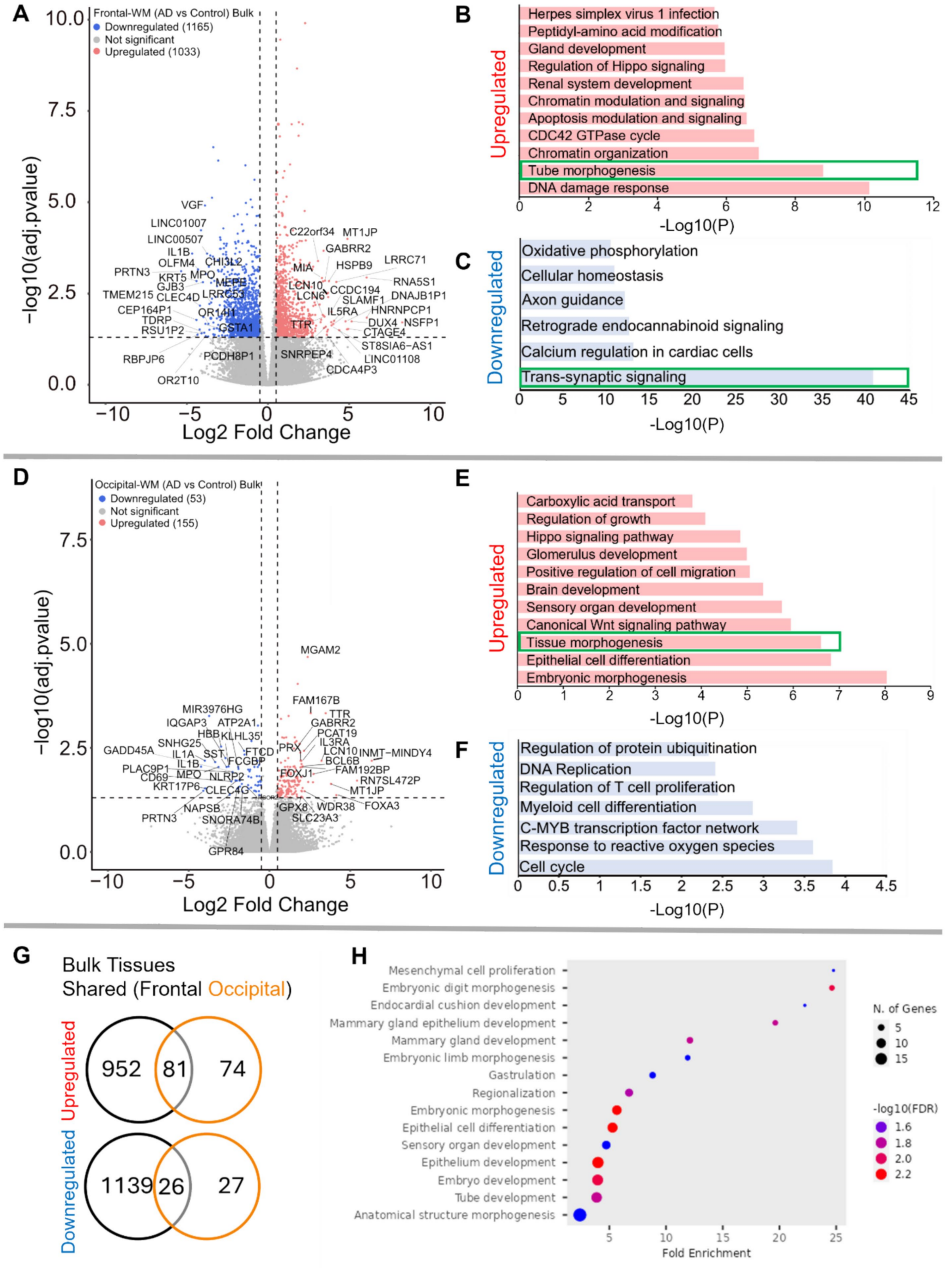
Gene expression changes in frontal-and occipital-WM bulk tissues. **(A,D)** Volcano plot showing differential gene expression in **(A)** frontal-WM bulk tissues and **(D)** occipital-WM bulk tissues. The color of dots indicates the direction of significantly altered genes, with red indicating upregulation and blue indicating downregulation. **(B,C,E,F)** Bar plot showing the enriched biological processes (GO/KEGG terms, canonical pathways) generated by Metascape for **(B,E)** upregulated genes, and **(C,F)** downregulated genes. The enriched pathways associated with brain vasculature in **(E,F)** and synaptic functions in **(C)** are marked by green boxes. **(G)** Venn diagram showing unique and shared DEGs in the frontal- and occipital-WM bulk tissues for upregulated genes (upper panel), and downregulated genes (lower panel). **(H)** Biological processes associated with upregulated genes common to frontal- and occipital-WM bulk tissues, generated by ShinyGO. The enriched pathways associated with brain vasculature **(B,E)** and synaptic functions in **(C)** are marked by green boxes.

We next sought to examine transcriptomic alterations unique to AD WM compared to controls, after confirming that the first 2–3 principal components of bulk WM gene expression data separate by disease status and not age or sex (Supplementary Figures S2A–D). In frontal-WM bulk tissues, RNA-seq identified 2,198 DEGs, of which 1,033 were significantly upregulated (log2FC > 0.5, BH-adjusted *p* < 0.05) in AD versus controls. Notable among upregulated genes are *RNA5S1, LRRC71, CCDC194, IL5RA, DUX4, CTAGE4, TTR* and *MLKL* (Figure 2A and Supplementary Table S1) and increased transcription of pseudogenes such as *NSFP1, MT1JP,* and *DNAJB1P1*. To examine the biological roles of the significantly upregulated genes, we performed functional gene enrichment analysis, which highlighted biological processes including DNA damage response, tube morphogenesis and regulation of Hippo-signaling (Figure 2B and Supplementary Table S2). Specifically, DNA damage included subclasses such as DNA metabolic process and DNA double strand break repair while tube morphogenesis encompasses blood vessel development, blood vessel morphogenesis, and angiogenesis (Supplementary Table S2).

Conversely, the 1,165 downregulated transcripts (log2FC < −0.5, BH-adjusted *p* < 0.05) in frontal-WM included *VGF, OLFM4, PRTN3, TMEM215, IL1B, KRT5, CHI3L2, GJB3, MPO,* and *GSTA1* (Figure 2A and Supplementary Table S1). Functional enrichment analysis of these significantly downregulated genes revealed pathways linked to trans-synaptic signaling, axon guidance, cellular homeostasis, and oxidative phosphorylation (Figure 2C and Supplementary Table S2). Representative trans-synaptic signaling genes include *SYT1, SYT2*, *SYT4*, *SYT5, SYT16, SYTL2*, *SYTL5*, *SNAP25*, *VAMP1*, and *OLFM4*. The enrichment of synaptic functions prompted us to further evaluate the synaptic location and functional GO of downregulated genes using SynGO, an experimentally annotated database for synaptic location and functional GO (49). None of the upregulated genes were enriched for synaptic components (Supplementary Figures S2B, S3A). By comparison, downregulated genes were highly enriched for transcripts known to be related to synaptic location (Supplementary Figure S3C) and function (Supplementary Figure S3D). Collectively, the differential gene expression observed in frontal-WM bulk tissue primarily indicates alterations in biological processes related to DNA damage, brain vasculature and synaptic function.

Examination of occipital-WM bulk tissues revealed only 208 DEGs between AD and control (Supplementary Table S3), with 155 upregulated transcripts including the genes *TTR, LCN10, MGAM2, FOXA3, FAM167B,* and *WDR38* (Figure 2D). Biological processes associated with these upregulated genes included epithelial cell differentiation and tissue morphogenesis (Figure 2E). Interestingly, subclasses within tissue morphogenesis showed many vasculature processes, including tube morphogenesis and regulation of endothelial cell migration (Supplementary Table S4). Moreover, we also observed enrichment of canonical Wnt-signaling and Hippo signaling pathways, which have been shown to regulate vascular networks and angiogenesis through endothelial cell proliferation and migration (50). This observation suggests that, as with frontal WM, vasculature processes are one among many notable altered biological processes in the occipital-WM.

Significantly downregulated genes in occipital-WM included *IL1A, IL1B, SNHG25, PRTN3, MPO,* and *SST* (Figure 2D). Functional enrichment annotation revealed that the downregulated genes in occipital-WM bulk tissues were linked to cell-cycle regulation and response to reactive oxygen species (ROS) (Figure 2F). Of note, none of the occipital-WM DEGs (either up- or down-regulated) were associated with synaptic processes (Supplementary Figures S3E,F).

To further compare WM transcriptome in these brain regions, we next examined unique and shared DEGs between frontal- and occipital-WM AD versus control bulk tissues. Although most altered genes in frontal-WM were unique, 52.3% of upregulated (81/155) and 49.0% of downregulated genes (26/53) in occipital-WM overlapped with frontal-WM bulk tissues (Figure 2G). Analysis of these shared upregulated genes using ShinyGO (v. 0.80) confirmed developmental pathways are universally upregulated in AD WM tissue (Figure 2H). Collectively, our findings from both frontal- and occipital-WM confirm that vasculature alterations are a notable shared feature of AD WM pathology while changes affecting WM synaptic functions are more regionally specific.

### Blood vessels from frontal- and occipital-WM upregulate protein folding genes

**Figure 3:**
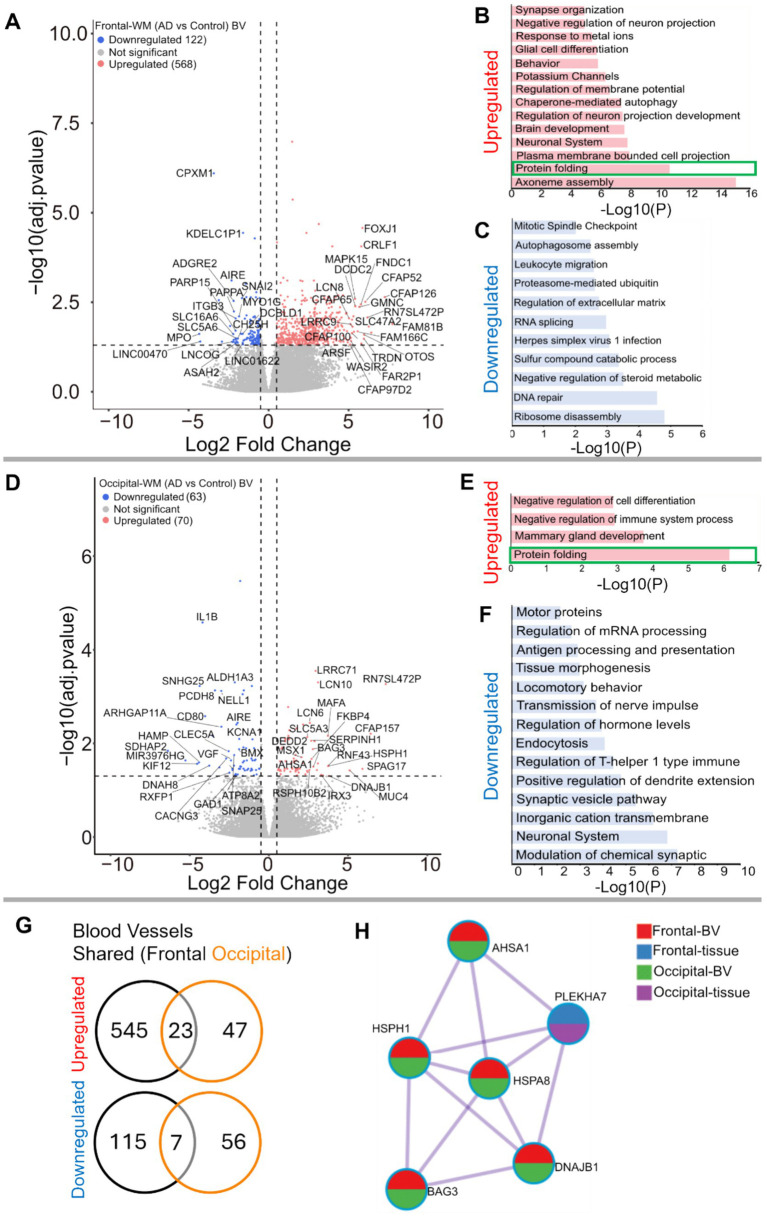
Gene expression changes in isolated blood vessels from frontal-and occipital-WM in AD cases. **(A,D)** Volcano plots showing differential gene expression in blood vessels (BV) of **(A)** frontal-WM and **(D)** occipital-WM. The color of dots indicates the direction of significantly altered genes, with red indicating upregulation and blue indicating downregulation. **(B,C,E,F)** Bar plot showing the enriched biological processes (GO/KEGG terms, canonical pathways) generated by Metascape for **(B,E)** upregulated genes, and **(C,F)** downregulated genes. Enriched pathways associated with protein folding in **(B,E)** are indicated by green boxes. **(G)** Venn diagram showing unique and shared DEGs in the isolated blood vessels from frontal- and occipital-WM for upregulated genes (upper panel) and downregulated genes (lower panel). **(H)** Enrichment network visualization for results from the significantly upregulated gene lists in AD versus control, color code represents that HSPs are generally shared among all RNA-seq samples.

Since both frontal- and occipital-WM showed shared gene expression changes related to brain vasculature function, we next investigated gene expression changes within isolated blood vessels. First, we confirmed that the isolated blood vessels were specifically enriched for endothelial cells, followed by vascular smooth muscle cells and pericytes (Supplementary Figures S4A,B). Differential expression analysis between AD and CTRL frontal-WM blood vessels then identified 690 DEGs. Of these, 568 were upregulated, including *LCN10, KLF15*, *FOXJ1, CRLF1, CFAP126, CFAP52, MAPK15, CFAP65, FNDC1, GMNC, SPAG17, FAM81B, FAM166C, LHX9, TRDN,* and *OTOS* (Figure 3A and Supplementary Table S5). Notable biological processes associated with these upregulated genes include axoneme assembly, protein folding, chaperone mediated autophagy, and response to metal ions (Figure 3B and Supplementary Table S6). Interestingly, protein folding emerged as one of the top altered biological processes after axoneme assembly. We also identified 122 downregulated transcripts in frontal-WM blood vessels, which were associated with multiple biological pathways including ribosome disassembly, RNA splicing, and regulation of extracellular matrix organization (Figure 3C).

Next, we examined if the protein folding pathway observed in frontal-WM blood vessels was also upregulated to occipital-WM blood vessels. Toward this goal, we first explored DEGs in occipital-WM blood vessels which yielded 133 total transcripts. Of these, 70 were upregulated including *LCN10, KLF15*, *CFAP157, SPAG17, MUC4, HSPH1, FKBP4,* and *HSPA8* (Figure 3D and Supplementary Table S7). Functional gene enrichment analysis showed that upregulated genes in occipital-WM blood vessels were most strongly associated with the protein folding pathway (Figure 3E and Supplementary Table S8). Last, in occipital-WM blood vessels, we observed 63 downregulated transcripts that were associated with the modulation of inorganic cation transport, endocytosis, and antigen processing (Figure 3F).

**Figure 4:**
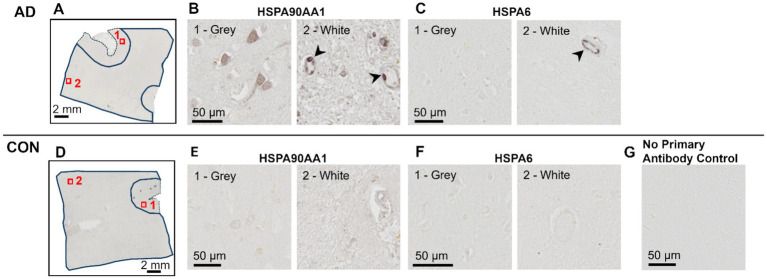
Histopathological validation of heat shock proteins. Immunohistochemistry of frontal-WM tissue from AD **(A–C)** and Control **(D–F)** donors. **(A,D)** An overview of tissue sections with gray matter (dashed line) and white matter (solid line) outlined showing the approximate location of higher magnification views shown in **(B,C,E,F)**. **(B,E)** HSP90AA1 and **(C,F)** HSPA6 labeling in an adjacent tissue section. Samples correspond to AD-8 and Control-5 (see Table 1). Arrowheads highlight vascular cells with heat shock protein positivity. **(G)** An image of tissue that was handled following the same procedure as in panels **(A–F)** but without the overnight incubation with primary antibody (experimental control).

While AD blood vessels isolated from frontal- and occipital-WM brain regions shared few up- or down-regulated transcripts (Figure 3G), we noted that at the pathway level, both brain regions were enriched for genes related to protein folding (Figures 3B,E). This enrichment included chaperone-mediated and *de novo*- protein folding, heat shock factor (HSF)-dependent transactivation, and protein maturation (Supplementary Tables S6, S8). We also observed more pronounced upregulation of heat shock proteins (HSPs) in blood vessels isolated from both frontal- and occipital-WM blood vessels than in bulk tissues (Figure 3H), which are one of the major components of the protein folding pathway (51, 52). To confirm the observed changes in the protein folding response of vascular cells, we performed histopathological validation of representative HSPs in sections from frontal-WM of donors used in this study. Consistent with the significant expression of *HSP90AA1* transcripts in blood vessels from AD frontal-WM (log2FC 2.98, padj. = 0.0091, Supplementary Table S5), HSP90AA1 protein was also expressed in AD frontal-WM blood vessels (Figures 4A,B). We also observed elevated gene expression of *HSPA6* (log2FC 4.83, padj. 0.0066, Supplementary Table S5) and confirmed protein expression by histology (Figure 4C). Notably, a similar extent of HSP-expressing vessels was absent from control tissues including donors with normal-appearing white matter and from tissue that was processed the same but not incubated with primary antibody – confirming specificity (Figures 4D–G). In sum, these observations indicate widespread proteostasis dysfunction in WM vasculature with AD pathology.

### Analysis of AD donors with high-WMH burden reveals distinct, regional upregulation of genes associated with immune function

**Figure 5:**
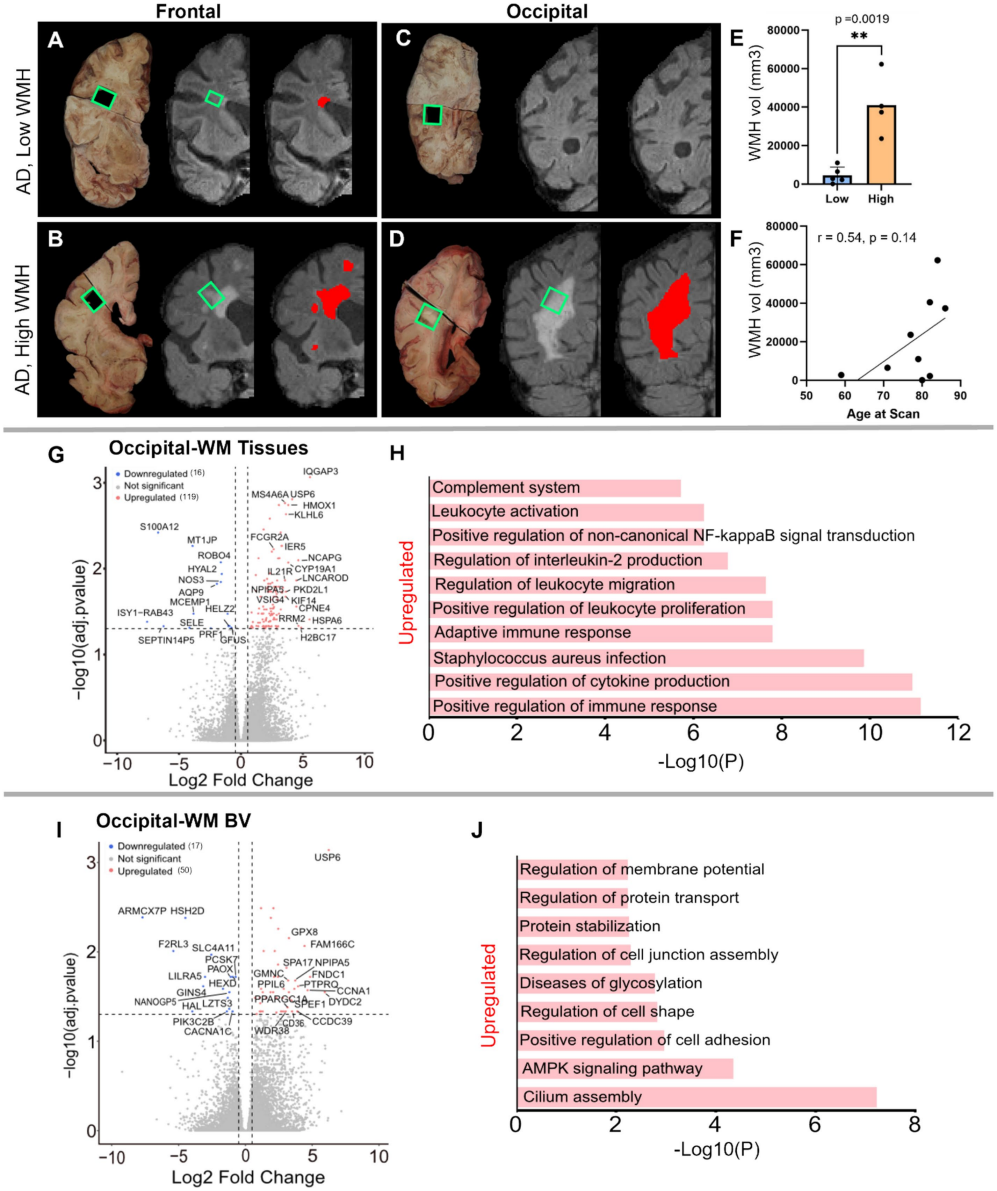
Characterization of low- and high-WMH in frontal- and occipital-WM in AD cases. **(A)** Examples of *in vivo* T2-weighted FLAIR MRI scans showing segmented WMH volumes (red pixels) in AD in frontal-WM **(A,B)** and occipital-WM **(C,D)**. Tissue sections indicated by green boxes corresponding to the *in vivo* WMH (red and gray pixels) from AD presenting low-WMH **(A,C)** and high-WMH **(B,D)** were dissected. **(E)** Comparison of high- and low-WMH samples based on volume of WMH region. **(F)** Pearson correlation between the *in vivo* MRI (volume) and age at the time of the scan. **(G–I)** Volcano plot showing differential gene expression in low- versus high-WMH in occipital-WM from **(G)** bulk tissues, and **(I)** blood vessels. **(H–J)** Bar plot showing the enriched biological processes (GO/KEGG terms, canonical pathways) generated by Metascape for low- versus high-WMH in occipital-WM from **(H)** bulk tissues, and **(J)** blood vessels.

Having characterized the transcriptomic differences in the tissues and blood vessels in AD relative to controls, we next explored gene expression differences in AD donors with low- or high-WMH burden. *A priori*, donors were classified into these groups based on *in vivo* MRI Fazekas scores (53) (Figures 5A–D; low = periventricular WMH ≤2, deep subcortical WMH ≤1; high = periventricular WMH >2, deep subcortical WMH >1). Comparison of total brain WMH volume measured from *in vivo* MRI revealed a significant difference between groups (*p*-value = 0.0019; Figure 5E): AD donors in the low-WMH group had WMH volumes of 100–11,000 mm^3^ while AD high-WMH donors ranged from 23,654 to 62,279 mm^3^. The average ages for low- and high-WMH AD patients were 77 ± 8.8 (*n* = 5) and 85 ± 5.4 (*n* = 4; Supplementary Figure S1B), respectively, and both groups had similar AD neuropathology (Braak V–VI and Thal 4–5 stages: Supplementary Figure S1B). Although WMH is widely recognized as a feature of aging that increases with age (2, 3, 54), we did not observe a statistically significant correlation between age and WMH volume in our sample (*p*-value 0.14) despite a positive correlation (Pearson *r* = 0.54) (Figure 5F). Given their similarities in age and AD pathology status, we next used samples from these donors to probe whether gene expression changes were further enhanced by WMH burden.

Differential gene expression analysis in the high- versus low-WMH groups yielded few transcripts in the frontal-WM after adjusting for multiple comparisons. In the frontal-WM bulk tissue, we found only two DEGs: *POLQ* (log2FC 2.8, padj. 0.01) and *BCAT2* (log2FC −0.6, padj. = 0.0005) (Supplementary Table S9). DNA polymerase theta (*POLQ*) has been shown to repair double-strand DNA breaks (55). Mitochondrial branched-chain amino-acid aminotransferase (*BCAT2*) is confined to vascular endothelial cells, where it triggers dysregulation of glutamate metabolism (56, 57). In blood vessels isolated from the frontal-WM, only 3 genes were upregulated (*NPIPA5, TPM3P9,* and *CWF19L2*) and two were downregulated (*MT-RNR1* and *PLPPR3*) (Supplementary Table S10). This suggests that transcriptional changes in frontal WM are not closely related to WMH burden.

By contrast, occipital-WM exhibited more DEGs in tissue (Figure 5G and Supplementary Table S11) and isolated blood vessels (Supplementary Table S12) with increasing WMH burden. We found 119 upregulated transcripts in occipital-WM bulk tissue including the genes *IQGAP3, HSPA6, H2BC17, RRM2, NCAPG, CPNE4, USP6, CYP19A1, HMOX1, KLHL6, PKD2L1, KIF14, IL21R* (Supplementary Table S11). Functional gene enrichment analysis showed that these upregulated genes in occipital WM bulk were predominantly associated with immune function encompassing cytokine production, adaptive immune response, leukocyte proliferation and migration, interleukin-2 production and complement system (Figure 5H and Supplementary Table S13). In blood vessels from occipital-WM, we found 50 upregulated transcripts including *USP6, DYDC2, FNDC1, CCNA1, FAM166C, PTPRQ, GRIN3B, CCDC39, NPIPA5, CLIC6, CD36* (Figure 5I and Supplementary Table S12). These upregulated genes were primarily associated with cilium assembly, AMPK signaling pathway, regulation of cell adhesion, and protein stabilization (Figure 5J and Supplementary Table S14). We also found 16 and 17 downregulated transcripts in occipital-WM bulk tissues and blood vessels respectively, which includes *NOS3, HYAL2, AQP9, and SELE* (tissue; Supplementary Table S11) and *CACNA1X, PAOX*, and *SLC4A11* (blood vessels; Supplementary Table S12). In sum, these data indicate that the extent of occipital WMH burden in individuals with AD is associated with greater inflammation, immune signaling, and cell adhesion.

## Discussion

White matter hyperintensities (WMH) are common age-associated neuroradiological changes associated with cognitive decline in Alzheimer’s disease (AD) (4, 17, 58–62). While WMHs are hypothesized to arise from vascular origins, the underlying molecular changes taking place in WMH-prone areas, and whether these differ between AD and aged, cognitively normal controls, are not well characterized. In this study we explored disease-related molecular features by RNA-seq comparison with age-matched cognitively intact controls and compared two spatially distant WM regions. We found notable overlap in gene expression programs related to blood vessels and protein folding pathways that are upregulated in WM with disease, but also noted key differences between regions. In particular, comparative analysis between AD groups with high- or low- WMH burden identified upregulation of pathways related to immune function and leukocyte activation particularly in the occipital-WM. Altogether, these data indicate that unique molecular pathways may drive WM changes in AD that are distinct from other age-related processes and that may differ even between WMH-susceptible brain regions.

Recent studies have shown that both the location (63) and severity (62) of WMHs across brain regions are associated with cognitive functions (64). Moreover, another study found that posterior-WMHs are linked to AD-related neurodegeneration, while anterior-WMHs are associated with both AD-related neurodegeneration and vascular mechanisms (65). These findings underscore the need to understand the biological mechanisms underlying WMHs throughout the brain. In our comparative analysis of two commonly affected WM brain regions, we found far more DEGs in the AD frontal-WM than occipital-WM control bulk tissues. Moreover, we found that genes associated with synapse function were significantly downregulated in AD frontal- but not occipital-WM bulk tissues. Synaptic structures in WM have previously been observed and may be related to modulation of oligodendrocytes and astrocytes, though the significance of their downregulation in AD remains to be characterized (66, 67). A similar involvement of synaptic input has also been observed in the cortical WM in human temporal lobe epilepsy (68), suggesting the involvement of synaptic dysregulation in WM. In sum, this highlights key differences in the molecular changes taking place between frontal- and occipital-WM in AD.

We also found that alterations in pathways associated with tube/tissue morphogenesis, vasculature development, and sprouting angiogenesis were upregulated in both frontal and occipital bulk WM tissues from AD donors. This is in line with the prevailing understanding that WMH changes are associated with vascular dysfunction (10). In addition, we found a few downregulated genes common to both brain regions, including *PRTN3, SST*, and *MPO*. Proteinase 3 (*PRTN3*) has been previously reported to regulate endothelial barrier function (69) whereas somatostatin (*SST*) has been shown to modulate cortical circuits and cognitive function (70). Myeloperoxidase (*MPO*) regulates oxidative stress, suggesting its downregulation may result in elevated ROS levels thus triggering the activation of stress response pathways (71). Closer examination of gene expression changes in blood vessels in frontal- and occipital-WM shows upregulated genes are associated with protein folding. The enrichment network visualization of upregulated genes from AD donors indicates that HSPs are generally shared among bulk tissues and blood vessels in both frontal- and occipital-WM brain regions, albeit with higher frequency in blood vessels (Figure 3H). The increased expression of genes associated with HSPs, which are a crucial part of the translational machinery and protein folding pathway, suggests that AD generally induces proteostatic stress in brain. This finding is consistent with previous reports of upregulated heat shock-related protein folding pathways in AD based single nucleus RNA-sequencing of endothelial cells from multiple gray matter cortical regions (33).

Interestingly, while comparison between AD and control donors yielded many DEGs, only two genes that were significantly upregulated across both regions in bulk tissue and blood vessels: lipocalin 10 (*LCN10*) and Krüppel-like factor 15 (*KLF15*). A recent study in mice highlights that LCN10 protects inflammation induced vascular leakage by regulating endothelial barrier integrity and permeability through an LRP2-Ssh1 mediated signaling pathway (72). Thus, the upregulation of *LCN10* suggests potential involvement in maintaining the vascular integrity in AD with WMHs. Likewise, *KLF15*, a zinc finger DNA-binding protein belonging to the Krüppel-like family of transcription factors, has also been shown to protect vascular endothelial dysfunction induced by tumor necrosis factor-alpha (TNF-*α*) in cultured human endothelial cells (73). Given this existing literature supporting the role of these genes in blood brain barrier dysfunction, this further supports that vasculature-related processes are key features of AD-related changes.

The transcriptomic analysis of the bulk tissue and blood vessels isolated from high-WMH in occipital-WM revealed that upregulated genes were related to the positive regulation of immune response, regulation of cell activation, positive regulation of cytokine production, and myeloid leukocyte activation. This is in line with a recent study demonstrating a correlation between leukocyte gene expression and WMH progression (74) and a recent case report indicating greater microglial burden in hyperintense WM areas compared to adjacent tissue (75). Moreover, another group employed epigenomic and integrative-omics data to identify 19 pivotal regulatory genes that influences WMH burden (76). These genes were associated with immune function, blood brain barrier, extracellular matrix organization, lipid, and lipoprotein metabolism (76). Another transcriptomic study demonstrated an augmented volume of WMH with the increased expression of 39 genes (67). Similarly, single-cell RNA sequencing of monocytes isolated from patients with cerebral small vessel disease showed an increased expression of cytokine production and pro-inflammatory markers (77). Consistent with our observations, comprehensive analysis of whole blood gene expression identified upregulation of cytokine-cytokine receptor interaction and B-cell receptor signaling (78). Our findings also agree with a recent epigenetics study that demonstrated DNA methylation is linked to WMH formation, particularly influencing blood–brain barrier function and immune response (79). Thus, the data presented here and elsewhere indicate that brain vasculature and immune functions are major altered pathways associated with WMH.

While this comprehensive RNA-seq study highlighted potential drivers of AD WMHs, there are limitations to this work that we hope to address with future studies. For one, given a small sample size and variations in gene expression among samples, a larger population-based cohort including diverse ethnic groups (80) may further substantiate the current findings. Second, our current samples were chosen based on the availability of *in vivo* MRI data. Consistent intervals between T2-FLAIR scans and death are difficult to obtain, and while we only selected cases with scans <5 years prior to tissue collection, we cannot exclude the possibility that some individuals who were classified as having a “low” WMH burden did not subsequently develop additional lesions (4, 74, 81, 82). Third, no *in vivo* MRI data was available to guide dissection of frozen tissues for RNAseq from the control tissues, precluding comparison between WMH in non-AD vs. AD individuals. Similarly, while we qualitatively examined WMH in all available tissues *ex vivo*, these images were acquired from the hemisphere contralateral to the ones used for RNA-seq per participant; despite our best attempt to match regions, it may not be possible to perform a one-to-one comparison between *in vivo* and *ex vivo* measures. In the future, these limitations might be overcome by pairing *ex vivo* MRI with spatial transcriptomics, which has reported compatibility with FFPE tissues and cellular resolution (83), in order to fully visualize molecular changes taking place both in and around WMH areas. Finally, variables other than WMH burden, such as sex, age, or genetic risk factors not assayed here, may further contribute to gene expression differences between AD donors and WMH burdens. In the future, additional histological examination of key gene targets described in individuals with WMH and AD would help confirm our findings and compare them to other neurological diseases.

In conclusion, our transcriptomic analysis underscores the role of brain vasculature processes and protein folding pathways as key contributors to global WM changes in the AD brain. While these features were shared between brain regions, additional gene expression changes were observed only in frontal-WM indicating separate processes are at work including downregulation of synaptic pathways in disease. Moreover, enhanced upregulation of cytokine production and immune functions were related to greater occipital WMH burden in individuals with AD providing insights as to how these lesions may contribute to faster cognitive decline in individuals with AD, though follow-up studies are warranted to confirm this finding.

## Data Availability

The datasets presented in this study can be found in online repositories. The names of the repository/repositories and accession number(s) can be found in the article/Supplementary material.

## Footnotes

^1^ https://metascape.org

^2^ http://bioinformatics.sdstate.edu/

^3^ https://www.syngoportal.org/
