# Supplementary material for: Molecular profiling of frontal and occipital subcortical white matter hyperintensities in Alzheimer’s disease
Source: Front Neurol. 2025 Jan 7;15:1470441. doi: 10.3389/fneur.2024.1470441 (PMC11753232; doi:10.3389/fneur.2024.1470441)
Supplement: Supplementary file 15 [file Supplementary_file_1.pdf]

Supplementary Material

Molecular profiling of frontal and occipital subcortical white matter hyperintensities in Alzheimer's disease.

Sulochan Malla[1,2,†], Annie G. Bryant[1,3,†], Benjamin Woost[1], Nina Wolf[1], Rojashree Jayakumar[1], Andrew Li[1], Sudeshna Das[1,2], Susanne J. van Veluw[1,2], Rachel E. Bennett[1,2,*]

[1] Department of Neurology, Massachusetts General Hospital, Charlestown, MA, USA
[2] Harvard Medical School, Boston, MA, USA
[3]School of Physics, The University of Sydney, Sydney, Australia
[†]These authors contributed equally to this work

* For correspondence

Department of Neurology, Massachusetts General Hospital, 114 16th Street, Charlestown, Massachusetts, USA; Tel: 617-726-1263, E-mail: rebennett@mgh.harvard.edu

Supplementary Data
The raw sequencing files are deposited in the NCBI Sequencing Read Archive (SRA) under the accession number PRJNA1186095.

Supplementary Tables

Sup. Table 1. Differentially Expressed Genes (DEGs) in Frontal-WM bulk tissue (2198 DEGs; 1033 up; 1165 down)

Sup. Table 2. Biological processes enriched in significantly up- or downregulated DEGs in Frontal-WM bulk tissues

Sup. Table 3. Differential Expressed Genes (DEGs) in Occipital-WM bulk tissue (208 DEGs; 155 up; 53 down)

Sup. Table 4. Biological processes enriched in significantly up- or downregulated DEGs in Occipital-WM bulk tissues

Sup. Table 5. Differentially Expressed Genes (DEGs) in Frontal-WM blood vessels (690 DEGs; 568 up; 122 down)

Sup. Table 6. Biological processes enriched in significantly up- or downregulated DEGs in Frontal-WM blood vessels

Sup. Table 7. Differentially Expressed Genes (DEGs) in Occipital-WM blood vessels (133 DEGs; 70 up; 63 down)

Sup. Table 8. Biological processes enriched in significantly up- or downregulated DEGs in Occipital-WM blood vessels

Sup. Table 9. High WMH vs. low WMH Differentially Expressed Genes (DEGs) in Frontal-WM bulk tissue

Sup. Table 10. High WMH vs. low WMH Differentially Expressed Genes (DEGs) in Frontal-WM blood vessels

Sup. Table 11. High WMH vs. low WMH Differentially Expressed Genes (DEGs) in Occipital-WM bulk tissue

Sup. Table 12. High WMH vs. low WMH Differentially Expressed Genes (DEGs) in Occipital-WM blood vessels

Sup. Table 13. Biological Processes enriched in significantly upregulated genes in high vs. low WMH occipital-WM bulk tissues

Sup. Table. 14. Biological processes enriched in significantly upregulated genes in high vs. low. WMH occipital-WM blood vessels

Supplementary Figures

A

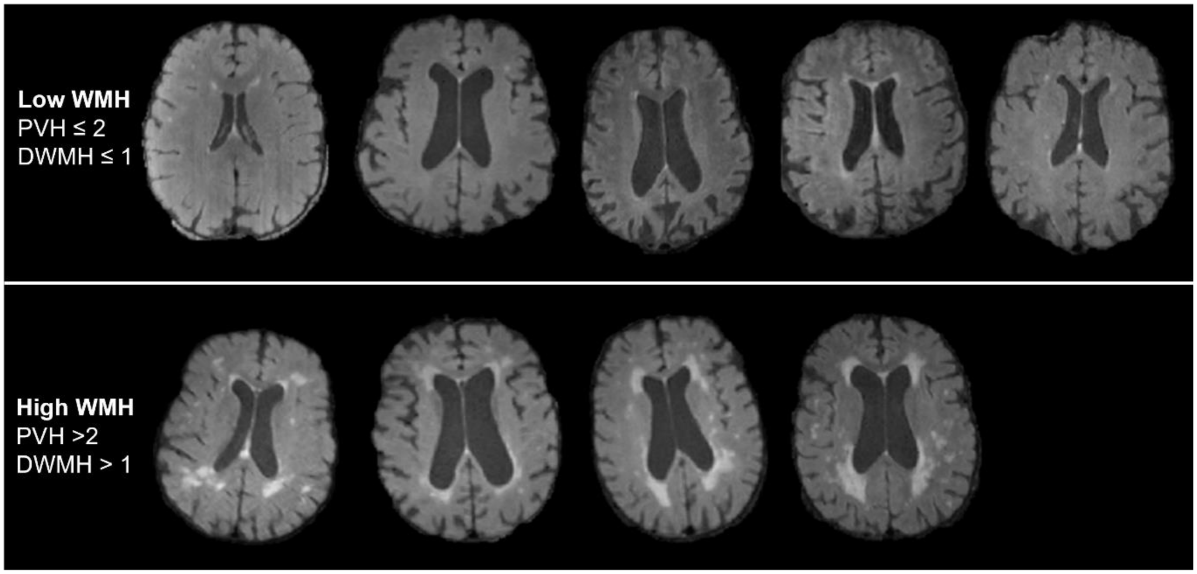

B

Group	Age (Mean ± STD)	Braak stage	Thal stage	Avg. pre-mortem scan int.
**Control (n=5)**	85 ± 5.1	I-II	0-3	NA
**AD, Low WMH (n=5)**	77 ± 8.8	V-VI	4-5	2.95 years
**AD, High WMH (n=4)**	85 ± 5.4	V-VI	4-5	2.81 years

Supplementary Figure 1. A) *In-vivo* T2-weighted FLAIR MRI showing the low (upper panel) and high (lower panel) WMH burden AD in donors (PVH- periventricular hyperintensities; DWMH = deep white matter hyperintensities). **B**) Information showing group averages for age, AD pathology (Braak and Thal stages), and pre-mortem scan interval.

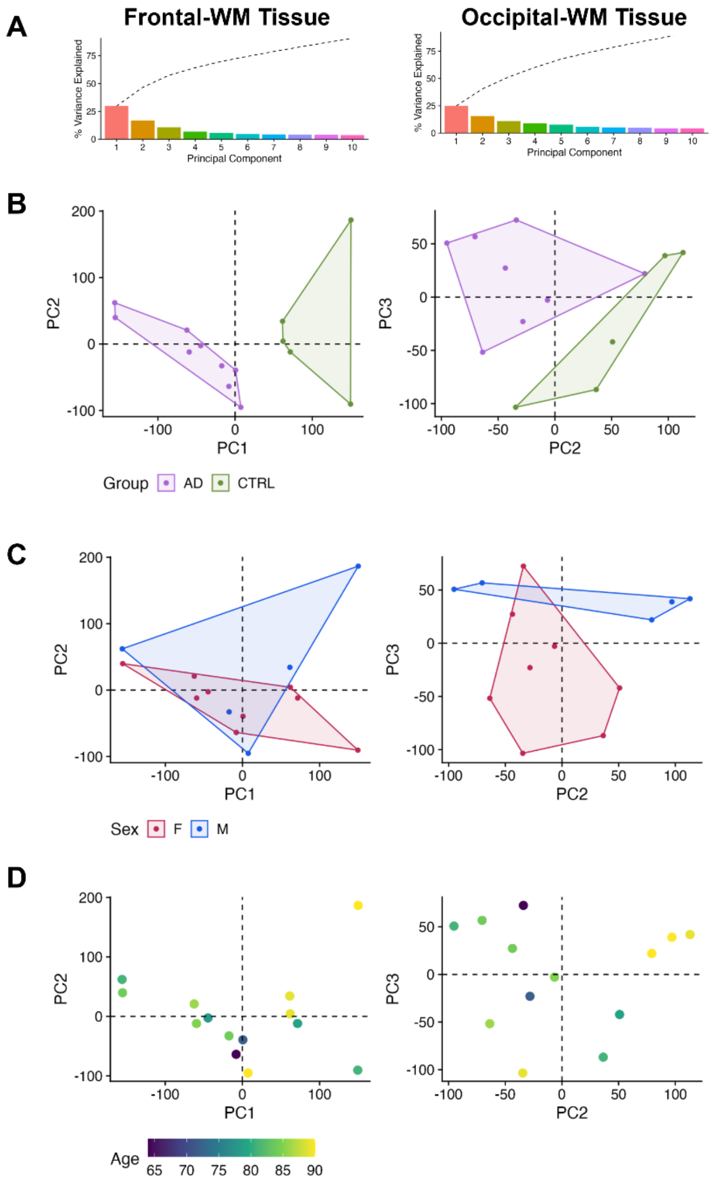

Supplementary Figure 2. A) Scree plots from PCA of bulk white matter tissue sample TPM count data showing the percent of variance explained by each of the principal components. The dashed line indicates the cumulative variance explained. **B)** The first two PCs from the frontal-WM cleanly separate AD (N=9) from control (CTRL; N=5) brains. While PC1 and PC2 did not cleanly separate CTRL from AD cases in the occipital-WM (data not shown), PC2 and PC3 scores in the occipital-WM could disentangle CTRL from AD cases. PC pairs that cleanly separated CTRL versus AD samples do not distinguish samples based on sex **(C)** or age **(D).**

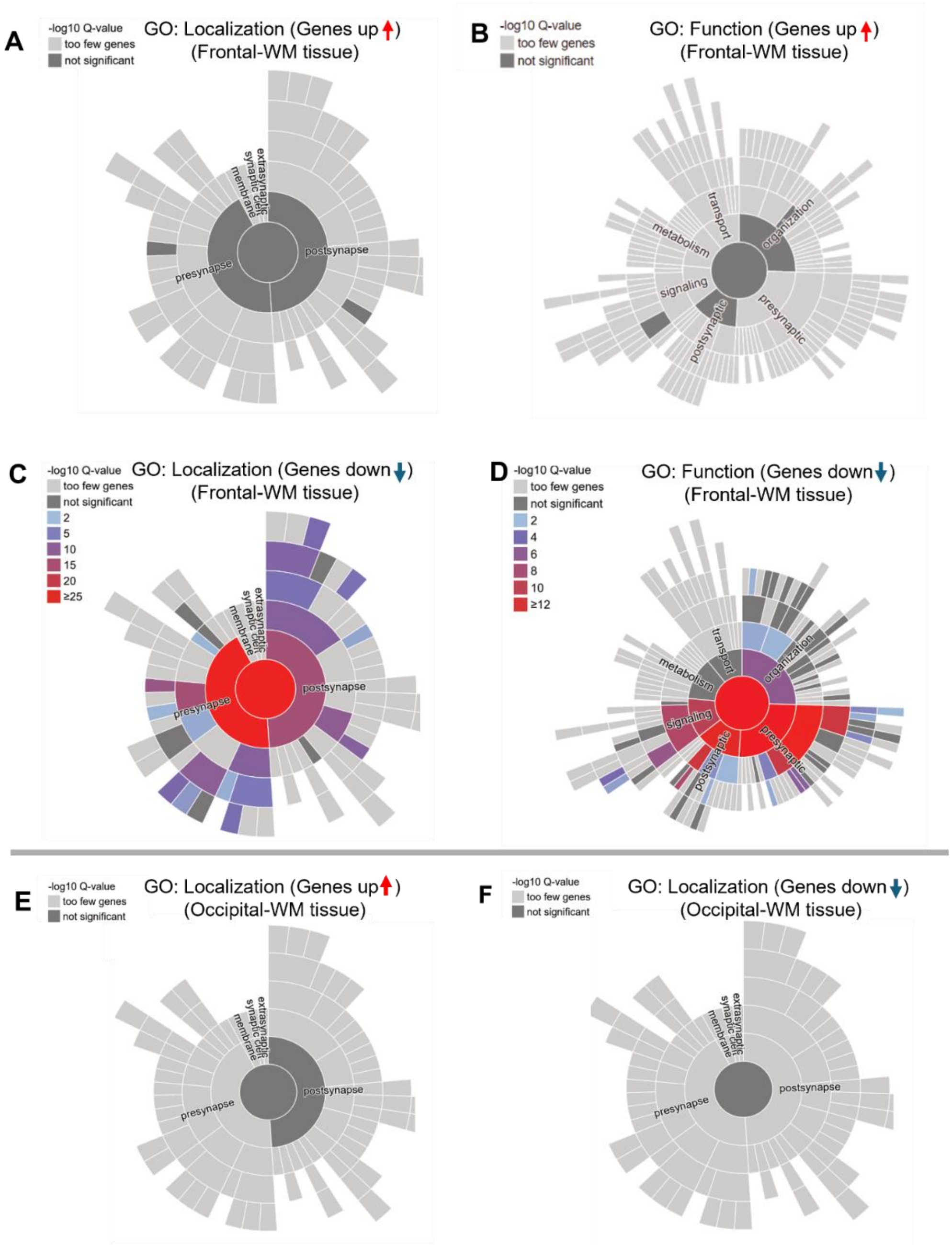

Supplementary Figure 3. A-B) Sunburst image of upregulated genes in frontal-WM bulk tissues in control versus AD with WMH, generated by SynGO showing **A)** cellular component enrichment, and (**B)** functional enrichment. **C-D**) Same as A & B but for downregulated genes in frontal-WM bulk tissues. **E-F**) Sunburst image showing the cellular component enrichment of (**E**) upregulated and (**F**) downregulated genes in occipital-WM bulk tissues. The red color in the center of the sunburst plot indicates the higher enrichment of significant synapse related genes while the light gray color suggests only a few genes are enriched in synaptic cellular component and dark gray color in the center of the sunburst plot indicates no significant enrichment of synapse related genes. The upward and downward pointed arrows refer to upregulated and downregulated genes, respectively.

A

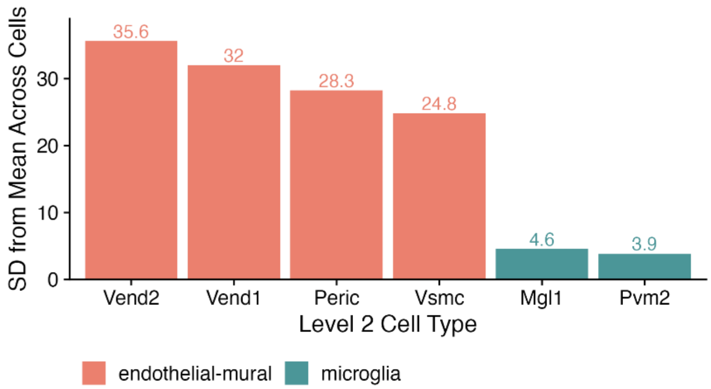

B

Level 2 Cell Type	BH-adjusted P	Fold Change	SD from mean
**Vend2**	**0**	**4.536138**	**35.617806**
**Vend1**	**0**	**4.985683**	**32.018011**
**Peric**	**0**	**4.226651**	**28.252179**
**Vsmc**	**0**	**4.002494**	**24.826463**
Mgl1	0.00096	1.65415	4.559383
Pvm2	0.0032	1.480894	3.87669

Supplementary Figure 4. Expression-weighted cell-type enrichment (EWCE) analysis confirms specific enrichment of vascular cells in the isolated blood vessels. **A)** For the six cell types with significant enrichment in the top 500 CTRL WM blood vessel DEGs (all BH-adjusted P<0.05), the standard deviations from the mean expression across all cell types are depicted. Bars are colored to indicate the broader cell type (endothelial-mural or microglia). **B)** The full set of statistics from EWCE are provided for the same six cell types depicted in **A**.
